# NF2 and ZFTA evaluation in the diagnostic algorithm of pediatric posterior fossa ependymoma with H3K27ME3 retained expression

**DOI:** 10.1186/s40478-023-01503-2

**Published:** 2023-01-13

**Authors:** Arnault Tauziède-Espariat, Yassine Ajlil, Marie-Anne Debily, David Castel, Jacques Grill, Stéphanie Puget, Lauren Hasty, Fabrice Chrétien, Alice Métais, Volodia Dangouloff-Ros, Nathalie Boddaert, Pascale Varlet

**Affiliations:** 1 Department of Neuropathology, GHU Paris-Psychiatrie et Neurosciences, Sainte-Anne Hospital. 1, Rue Cabanis, 75014 Paris, France; 2 U981, Molecular Predictors and New Targets in Oncology, INSERM, Gustave Roussy, Université Paris-Saclay, 94805 Villejuif, France; 3 Univ. Evry, Université Paris-Saclay, 91000 Evry, France; 4 Department of Pediatric Oncology, Gustave Roussy, Université Paris-Saclay, 94805 Villejuif, France; 5 Department of Pediatric Neurosurgery, Necker Hospital, APHP, Université Paris Descartes, Sorbonne Paris Cite, 75015 Paris, France; 6 Pediatric Radiology Department, AP-HP, Hôpital Universitaire Necker-Enfants Malades, 75015 Paris, France; 7 Université Paris Cité, INSERM U1299, 75015 Paris, France; 8 Université Paris Cité, UMR 1163, Institut Imagine, 75015 Paris, France

Posterior fossa ependymomas can be subdivided into two types based on molecular profiling: A (PFA) and B (PFB) [4]. Immunophenotypically, they differ in the expression of H3K27me3: PFA show loss of this mark while PFB retain its expression. Furthermore, they also differ in the age of onset (pediatric *vs.* adult in PFA and PFB, respectively) [5]. However, because the maintainance of H3K27me3 immunoexpression is not pathognomonic, the most recent World Health Organization (WHO) classification has defined that a diagnosis of PFB may be rendered using DNA-methylation profiling [4]. Moreover, rare posterior fossa ependymomas with *ZFTA* fusion, exhibiting a preserved expression of H3K27me3, have been reported in pediatric cases [2]. To date, no PFB pediatric series has been reported and only epigenetic data are available in the literature [1]. The aim of this study was to clinically, radiologically, and molecularly (including DNA-methylation profiling) characterize a retrospective series of pediatric PFB (diagnosed solely by histopathology and immunohistochemistry) to detect potential differential diagnoses.

This pediatric series included 15 tumors initially diagnosed as PFB before the 2021 WHO guidelines, based on retained immuno-expression of H3K27me3. Subependymomas were excluded. The children were aged 1 to 17 years-old (median age: 11) with a female predominance (sex ratio: 1.5). A central neuroradiological review confirmed that all tumors were located in the posterior fossa. The DNA-methylation profiling classified tumors into four groups: PFB (n = 9), PFA (with low calibrated scores) (n = 2), supratentorial ependymomas, ZFTA-RELA fusion positive (n = 2), and spinal ependymomas (n = 2) (Table 1 and Additional files 1, 2, 3, 4 for details). We also performed a t-SNE (t-distributed stochastic neighbor embedding) analysis to better classify tumors with low calibrated scores (< 0.9) (Fig. 1). Using t-SNE, one of the two cases (#10) classified as PFA was reclassified as PFB whereas the other (#11), which presented a heterogeneous staining for H3K27me3 (without any positivity for EZHIP and H3K27M) was in close vicinity with PFA. The two cases classified as ependymomas, ZFTA-RELA fusion positive were confirmed by immunohistochemistry (L1CAM and NFκB immunopositivities), and RNA sequencing analysis (*ZFTA::RELA* fusion). Central neuroradiological review confirmed that both tumors were located in the posterior fossa without a supratentorial component. Interestingly, both cases presented clinical and radiological similarities and were distinct from PFB: they concerned the youngest patients (aged 1 and 3 years-old) and were revealed by a solid tumor located in the upper part of the fourth ventricle towards the aqueduct (compared to other PFB from the cohort which were located in the lower part of the fourth ventricle and crossed the Magendie foramen). The two cases classified as spinal ependymomas were located in the medulla oblongata or at the bulbo-medullar junction in a context of neurofibromatosis type 2 (NF2).

**Fig. 1:**
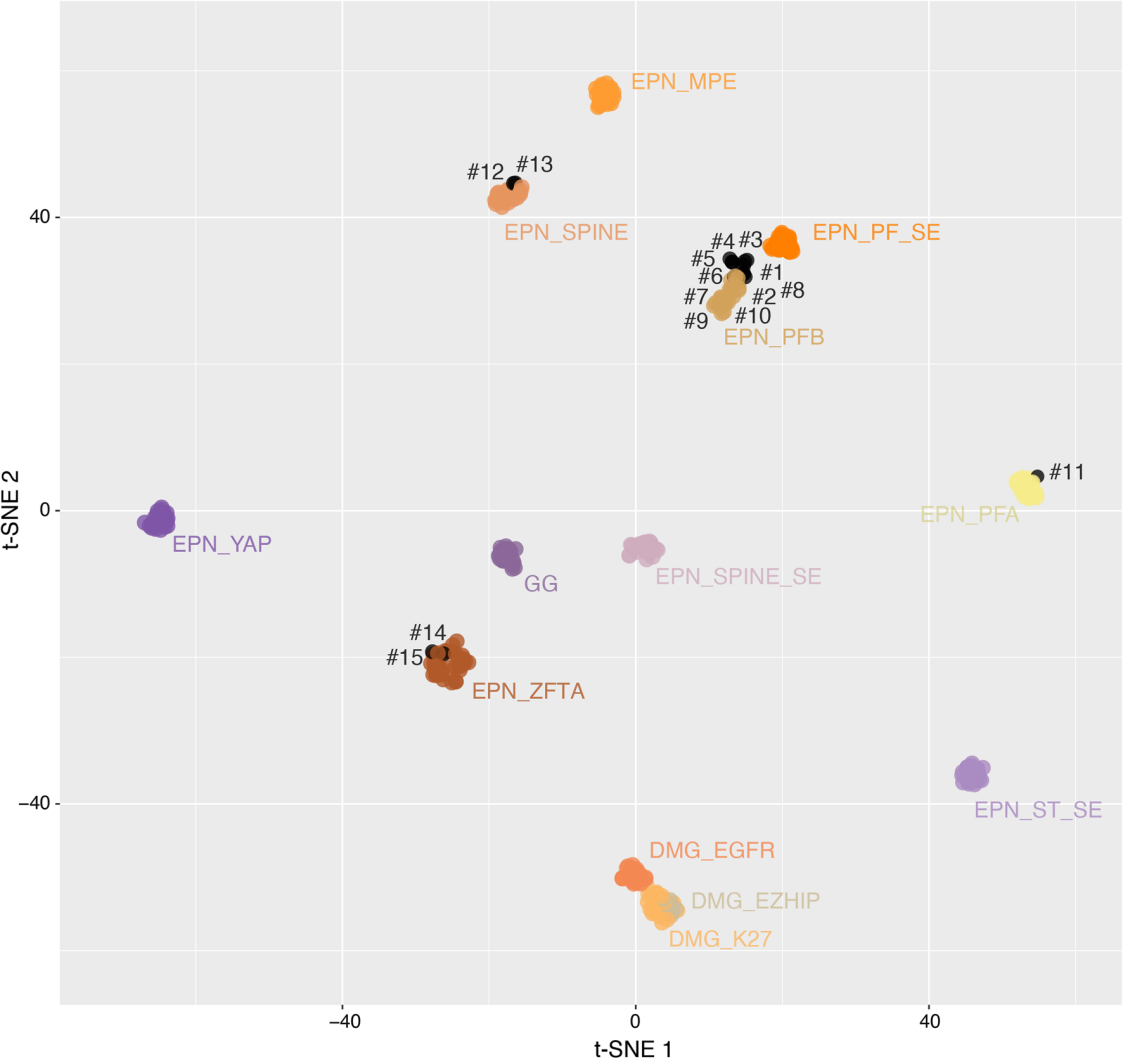
t-distributed stochastic neighbor embedding analysis of DNA methylation profiles of the investigated tumors alongside selected reference samples. Reference DNA methylation classes: diffuse midline glioma H3 K27M mutant (DMG_K27), diffuse midline glioma *EGFR*_altered (DMG_EGFR), diffuse midline glioma *EZHIP*_overexpressed (DMG_EZHIP), low-grade glioma, ganglioglioma (GG), ependymoma, myxopapillary (EPN_MPE), ependymoma, posterior fossa group A (EPN_PFA), ependymoma, posterior fossa group B (EPN_PFB), ependymoma, ZFTA fusion (EPN_ZFTA), ependymoma, spinal (EPN_SPINE), ependymoma, YAP fusion (EPN_YAP), subependymoma, posterior fossa (EPN_PF_SE), subependymoma, spinal (EPN_SPINE_SE), subependymoma, supratentorial (EPN_ST_SE)

**Table 1:** Summary of clinicopathological features of the cohort

Case	Age (years)	Sex	H3K27me3	NFKB	L1CAM	Molecular analysis	1q gain	Methylation class (calibrated score)	Resection	Metastasis at diagnosis	Adjuvant therapies	Outcome
1	10	M	Preserved	Negative	Negative	NA	Absent	EPN-PFB (0.90) SUBCLASS 3 (0.64)	TR	No	No	Relapse-free. Alive at 79 months
2	11	M	Preserved	Negative	Negative	NA	Absent	EPN-PFB (0.30)	STR	No	RT	Relapse-free. Alive at 29 months
3	15	F	Preserved	Negative	Negative	NA	Absent	EPN-PFB (0.99) SUBCLASS 2 (0.86)	TR	No	RT	Relapse-free. Alive at 76 months
4	17	F	Preserved	Negative	Negative	NA	Absent	EPN-PFB SUBCLASS 1 (0.98)	TR	No	RT	Relapse-free. Alive at 22 months
5	11	M	Preserved	Negative	Negative	NA	Absent	EPN-PFB SUBCLASS 1 (0.99)	TR	No	RT	Relapse-free. Alive at 7 months
6	14	F	Preserved	Negative	Negative	NA	Absent	EPN-PFB SUBCLASS 2 (0.96)	TR	Yes, dural	RT	Relapse-free. Alive at 25 months
7	11	F	Preserved	Negative	Negative	NA	Absent	EPN-PFB SUBCLASS 4 (0.99)	TR	No	RT	Relapse-free. Alive at 126 months
8	11	F	Preserved	Negative	Negative	NA	Absent	EPN-PFB SUBCLASS 4 (0.99)	TR	No	RT	Relapse-free. Alive at 90 months
9	13	M	Preserved	Negative	Negative	NA	Absent	EPN-PFB SUBCLASS 4 (0.99)	TR	No	RT	Relapse-free. Dead at 127 months after rhabdomyosarcoma and melanoma
10	5	M	Preserved	Negative	Negative	NA	Absent	EPN-PFA SUBCLASS 1A (0.25)	TR	No	No	Local relapse at 14 months. Alive at 14 months
11	15	F	Heterogeneous	Negative	Negative	NA	Present	EPN-PFA SUBCLASS 1C (0.53)	PR	No	RT	Supratentorial relapse at 16 months. Alive at 16 months
12	15	F	Preserved	Negative	Negative	NF2	Absent	SPINAL EPN (0.99)	TR	No	No	Relapse-free. Alive at 63 months
13	12	F	Preserved	Negative	Negative	NF2	Absent	SPINAL EPN (0.99)	TR	No	CT + RT	Local relapse at 22 months. Alive at 98 months
14	3	F	Preserved	Positive	Positive	*ZFTA::RELA*	Absent	ST-EPN ZFTA-RELA SUBCLASS A (0.97)	STR	No	RT	Distant leptomeningeal relapse at 12 months. Alive at 39 months
15	1	M	Preserved	Positive	Positive	*ZFTA::RELA*	Absent	ST-EPN ZFTA-RELA SUBCLASS A (0.99)	PR	No	CT + RT	Local relapse at 8 months. Alive at 114 months

*CT* chemotherapy, *EPN* ependymoma, *F* female, *M* male, *NA* not available, *PFA* posterior fossa group A, *PFB* posterior fossa group B, *PR* partial resection, *RT* radiation therapy, *STR* subtotal resection, *TR* total resection

Herein, the integrative histopathological, genetic and epigenetic analyses, including t-SNE (Fig. 1) segregated tumors into: 10 PFB (66.7%, with an enrichment of subclass 4 as previously reported [1]), two ependymomas, *ZFTA-*fusion positive (13.3%), and two NF2-associated spinal ependymomas (13.3%). The last case (#11) remained not elsewhere classified (NEC) posterior fossa ependymoma (6.7%) because of the discrepancy between immunohistochemistry and DNA-methylation profiling (performed two times). Further similar EPN, NEC cases are needed to clarify their classification and the existence of other potential diagnostic biomarkers. Previously, infratentorial (one cervicomedullary and two cerebellar) ependymomas, *ZFTA-*fusion positive (one *ZFTA::MAML2*, one *ZFTA::NCOA2*, and one *ZFTA::RELA* fusion) were reported in children aged 3, 4 and 11 years [2]. Like our cases, the DNA-methylation profiling confirmed that they clustered with their supratentorial counterpart *ZFTA-*fused [2]. Our results reinforce that *ZFTA* fusion can occur in the posterior fossa, and therefore, constitutes a potential diagnostic pitfall. In these conditions, L1CAM and NFκB immunostainings may represent useful diagnostic biomarkers in the detection of *ZFTA-*fused cases. Interestingly, *ZFTA-*fusion positive posterior fossa ependymomas seem to occur more frequently in younger patients than pediatric PFB, which are very rare before adolescence [1]. Similarly to a previously reported case, our series highlights an epigenetic distinction between spinal ependymomas centered in the medulla and PFB ependymomas, suggesting distinct cellular origins [3]. This precise anatomical site is well described in NF2 in which almost all ependymomas occurr in the cervicomedullary junction [3].

In conclusion, the results of this pediatric series are in line with the newly established essential diagnostic criteria for PFB in the WHO classification. A diagnosis of pediatric PFB ependymoma cannot be proposed solely based on the retention of H3K27me3 immunoexpression, as they encompass at least three different histomolecular tumoral types. Our study highligths also the importance of the integration of clinical, radiological, and neuropathological data to achieve an accurate diagnosis. According to our results, it may be recommended to perform L1CAM and NFκB immunohistochemistry and to search for clinical and radiological criteria for NF2 before performing DNA-methylation profiling for the differential diagnosis of pediatric PFB ependymoma.

## Supplementary Information

**Additional file 1: Fig. S1.** Radiological and histomolecular features of reclassified posterior fossa ependymomas as spinal ependymomas Case 12 (**A**–**E**): **A**–**C** IRM in a NF2 patient showing bilateral vestibular schwannomas, multiple meningiomas (**B**, **C**) and a median intra-parenchymal mass with high contrast enhancement in the bulbo-medullary junction. **D** Ependymoma with tanycytic features (HPS, 40 × magnification). **E** H3K27me3 immunopositivity in the tumor cells (40 × magnification). Case 13: **F** IRM showing a large median mass originating from the medulla oblongata and exophytic in the fourth ventricle with a heterogeneous enhancement after injection of gadolinium. **G** Heterogeneous intensity on T2-weighted image. **H** Ependymal proliferation with pseudorosettes (HPS, 40 × magnification). **I** H3K27me3 immunopositivity in the tumor cells (40 × magnification). HPS: hematoxylin, phloxin and saffron. Black scale bars represent 50 μm.

**Additional file 2: Fig. S2.** Radiological and histomolecular features of posterior fossa ependymomas, ZFTA-fusion positive. Case 14: **A** IRM showing median mass located in the upper part of the fourth ventricle towards the aqueduct on T1-weighted image. **B** Heterogeneous signal on T2-weighted image. **C** Ependymal proliferation with pseudorosettes (HPS, 40 × magnification). **D** H3K27me3 immunopositivity in the tumor cells (40 × magnification). **E** Nuclear NFκB immunoexpression by tumor cells (40x magnification). Case 15: **F** IRM showing a median mass located in the upper part of the fourth ventricle towards the aqueduct on a T2-weighted image. **G** Heterogeneous enhancement after injection of gadolinium. **H** Highly cellular ependymal proliferation (HPS, 40 × magnification). **I** H3K27me3 immunoreactivity in the tumor cells (40 × magnification). **J** NFκB expression by tumor cells (40 × magnification). **K** RNAseq analysis highlights a fusion between ZFTA and RELA genes in each case, with a breakpoint at the exon 3 and 2 for ZFTA and at the exon 2 for RELA. HPS: hematoxylin, phloxin and saffron. Black scale bars represent 50 μm.

**Additional file 3: Fig. S3.** Immunohistochemical features of the posterior fossa ependymoma, not elsewhere classified. Case 11: **A** Heterogeneous expression of H3K27me3 (40 × magnification). **B** No immunoexpression for EZHIP (40 × magnification). Black scale bars represent 50 μm.

**Additional file 4: Fig. S4.** Uniform Manifold Approximation and Projection for Dimension Reduction (UMAP) analysis of DNA methylation profiles of the investigated tumors alongside selected reference samples Reference DNA methylation classes: diffuse midline glioma H3 K27M mutant (DMG_K27), diffuse midline glioma EGFR_altered (DMG_EGFR), diffuse midline glioma EZHIP_overexpressed (DMG_EZHIP), low-grade glioma, ganglioglioma (GG), ependymoma, myxopapillary (EPN_MPE), ependymoma, posterior fossa group A (EPN_PFA), ependymoma, posterior fossa group B (EPN_PFB), ependymoma, ZFTA fusion (EPN_ZFTA), ependymoma, spinal (EPN_SPINE), ependymoma, YAP fusion (EPN_YAP), subependymoma, posterior fossa (EPN_PF_SE), subependymoma, spinal (EPN_SPINE_SE), subependymoma, supratentorial (EPN_ST_SE).
