# Supplementary figures and images for: NF2 and ZFTA evaluation in the diagnostic algorithm of pediatric posterior fossa ependymoma with H3K27ME3 retained expression
Source: Acta Neuropathol Commun. 2023 Jan 13;11:9. doi: 10.1186/s40478-023-01503-2 (PMC9837912; doi:10.1186/s40478-023-01503-2)

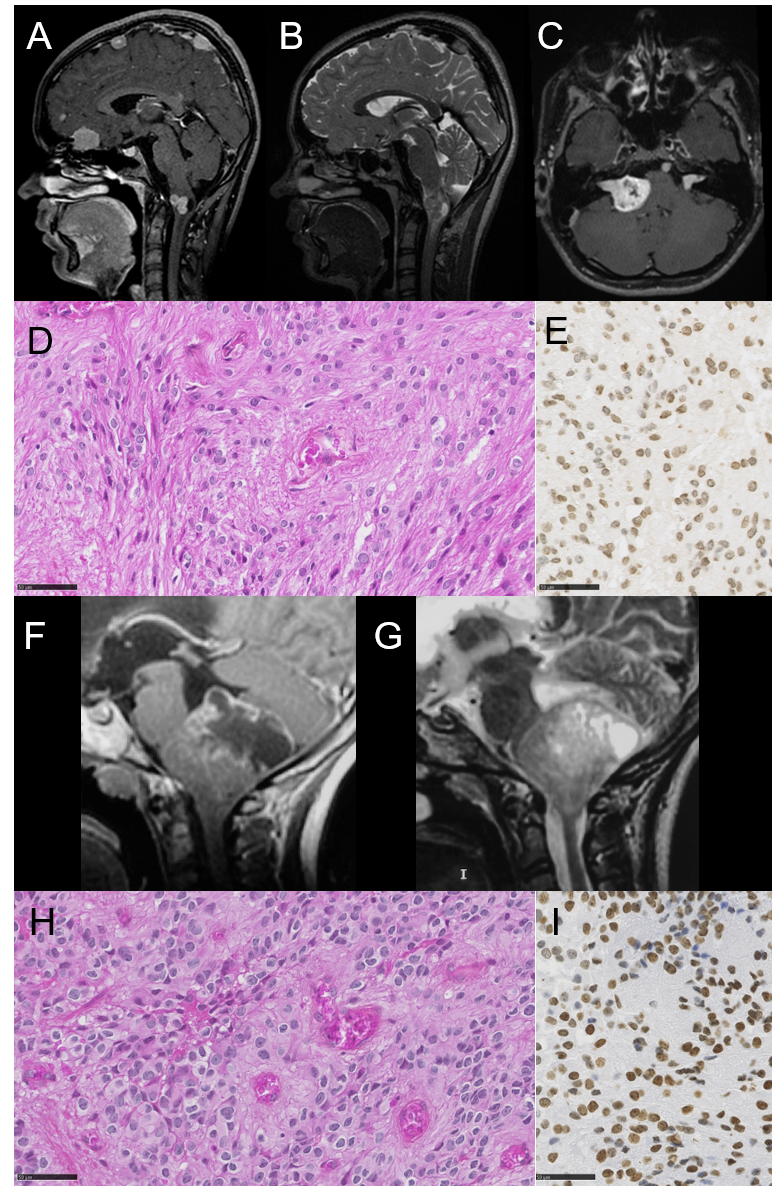

Supplement: Supplementary file 1 — Additional file 1: Fig. S1. Radiological and histomolecular features of reclassified posterior fossa ependymomas as spinal ependymomas Case 12 (A–E): A–C IRM in a NF2 patient showing bilateral vestibular schwannomas, multiple meningiomas (B, C) and a median intra-parenchymal mass with high contrast enhancement in the bulbo-medullary junction. D Ependymoma with tanycytic features (HPS, 40 × magnification). E H3K27me3 immunopositivity in the tumor cells (40 × magnification). Case 13: F IRM showing a large median mass originating from the medulla oblongata and exophytic in the fourth ventricle with a heterogeneous enhancement after injection of gadolinium. G Heterogeneous intensity on T2-weighted image. H Ependymal proliferation with pseudorosettes (HPS, 40 × magnification). I H3K27me3 immunopositivity in the tumor cells (40 × magnification). HPS: hematoxylin, phloxin and saffron. Black scale bars represent 50 μm. [file 40478_2023_1503_MOESM1_ESM.tif]

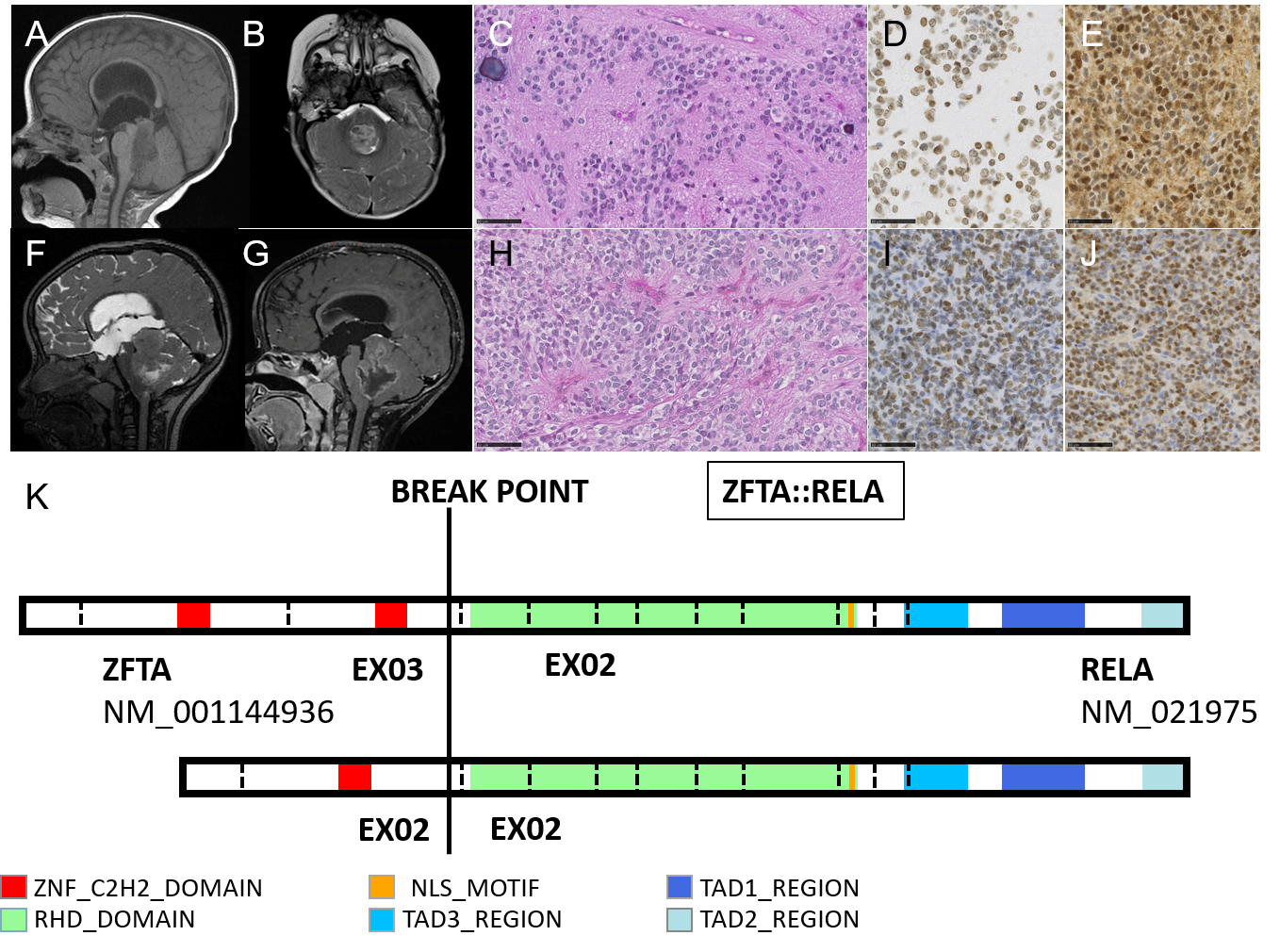

Supplement: Supplementary file 2 — Additional file 2: Fig. S2. Radiological and histomolecular features of posterior fossa ependymomas, ZFTA-fusion positive. Case 14: A IRM showing median mass located in the upper part of the fourth ventricle towards the aqueduct on T1-weighted image. B Heterogeneous signal on T2-weighted image. C Ependymal proliferation with pseudorosettes (HPS, 40 × magnification). D H3K27me3 immunopositivity in the tumor cells (40 × magnification). E Nuclear NFκB immunoexpression by tumor cells (40x magnification). Case 15: F IRM showing a median mass located in the upper part of the fourth ventricle towards the aqueduct on a T2-weighted image. G Heterogeneous enhancement after injection of gadolinium. H Highly cellular ependymal proliferation (HPS, 40 × magnification). I H3K27me3 immunoreactivity in the tumor cells (40 × magnification). J NFκB expression by tumor cells (40 × magnification). K RNAseq analysis highlights a fusion between ZFTA and RELA genes in each case, with a breakpoint at the exon 3 and 2 for ZFTA and at the exon 2 for RELA. HPS: hematoxylin, phloxin and saffron. Black scale bars represent 50 μm. [file 40478_2023_1503_MOESM2_ESM.tif]

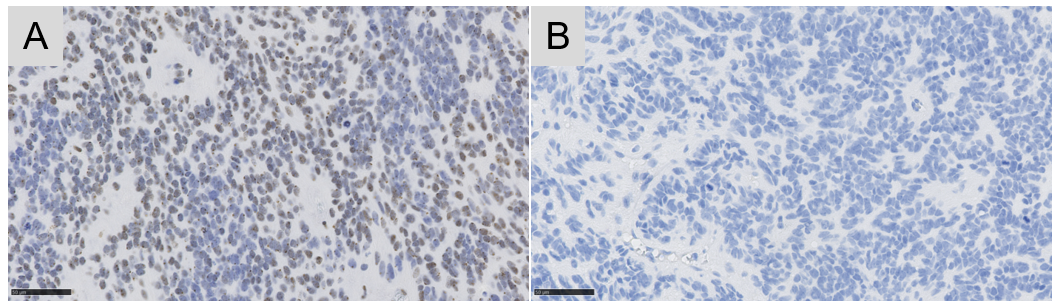

Supplement: Supplementary file 3 — Additional file 3: Fig. S3. Immunohistochemical features of the posterior fossa ependymoma, not elsewhere classified. Case 11: A Heterogeneous expression of H3K27me3 (40 × magnification). B No immunoexpression for EZHIP (40 × magnification). Black scale bars represent 50 μm. [file 40478_2023_1503_MOESM3_ESM.tif]
